# VariantSurvival: a tool to identify genotype–treatment response

**DOI:** 10.3389/fbinf.2023.1277923

**Published:** 2023-10-11

**Authors:** Thomas Krannich, Marina Herrera Sarrias, Hiba Ben Aribi, Moustafa Shokrof, Alfredo Iacoangeli, Ammar Al-Chalabi, Fritz J. Sedlazeck, Ben Busby, Ahmad Al Khleifat

**Affiliations:** ^1^ Genome Competence Center (MF1), Robert Koch Institute, Berlin, Germany; ^2^ Computational Mathematics Division, Department of Mathematics, Stockholm University, Stockholm, Sweden; ^3^ Faculty of Science of Tunis, University El Manar, Tunis, Tunisia; ^4^ Department of Computer Science, University of California, Davis, CA, United States; ^5^ Maurice Wohl Clinical Neuroscience Institute, King’s College London, London, United Kingdom; ^6^ Human Genome Sequencing Center, Baylor College of Medicine, Houston, TX, United States; ^7^ DNAnexus, Mountain View, CA, United States

**Keywords:** structural variants, survival analysis, clinical trials, personalized medicine, Kaplan–Meier, Cox regression, R shiny

## Abstract

**Motivation:** For a number of neurological diseases, such as Alzheimer’s disease, amyotrophic lateral sclerosis, and many others, certain genes are known to be involved in the disease mechanism. A common question is whether a structural variant in any such gene may be related to drug response in clinical trials and how this relationship can contribute to the lifecycle of drug development.

**Results:** To this end, we introduce VariantSurvival, a tool that identifies changes in survival relative to structural variants within target genes. VariantSurvival matches annotated structural variants with genes that are clinically relevant to neurological diseases. A Cox regression model determines the change in survival between the placebo and clinical trial groups with respect to the number of structural variants in the drug target genes. We demonstrate the functionality of our approach with the exemplary case of the *SETX* gene. VariantSurvival has a user-friendly and lightweight graphical user interface built on the shiny web application package.

## 1 Introduction

Neurodegenerative disorders, such as Alzheimer’s disease, Parkinson’s disease, and amyotrophic lateral sclerosis, impose a substantial burden on patients, caregivers, and healthcare systems worldwide (GBD, 2019 Dementia Forecasting Collaborators, 2022; Feigin et al., 2020). Despite decades of research, the underlying mechanisms driving disease progression and variability in patient survival remain incompletely understood (Martin et al., 2017; Robinson et al., 2023). Genetic factors have been increasingly recognized as crucial contributors to the pathogenesis and progression of neurodegeneration. Recent advancements in genomic technologies have enabled the identification of various genetic markers, including single-nucleotide variants, copy number variations, and structural variations, that may play significant roles in disease susceptibility, progression, and prognosis (Langbehn et al., 2019; van Rheenen et al., 2021; Al Khleifat et al., 2022).

An accurate evaluation of patient survival is a critical aspect of clinical trials aimed at assessing the efficacy of therapeutic interventions for neurodegenerative diseases. Traditional survival analysis methods, such as Kaplan–Meier curves (Kaplan and Meier, 1958) and Cox proportional hazards models (Cox, 1972; T. M. Therneau and Grambsch, 2000), have been widely used. However, these approaches often overlook the intricate genetic landscape underlying disease heterogeneity, limiting their ability to capture the full extent of genetic influences on patient survival (Willemse et al., 2022). Thus, there is a compelling need for methodologies that integrate genetic markers to enhance the precision and predictive power of survival assessments in neurodegenerative clinical trials.

Structural variations (SVs) are genomic alterations involving large-scale rearrangements, including deletions, duplications, inversions, and translocations. Emerging evidence suggests that SVs can profoundly impact gene expression and disrupt regulatory elements associated with neurodegeneration (Marshall et al., 2008; Lupski et al., 2010; Carvalho and Lupski, 2016; Marshall et al., 2017). Consequently, these genomic alterations hold substantial potential as prognostic indicators of disease progression and survival outcomes in clinical trials. We have previously shown the correlation between structural variants and survival in neurodegenerative diseases such as amyotrophic lateral sclerosis and frontotemporal dementia, highlighting the importance of testing structural variants in clinical trials (Al Khleifat et al., 2022). Understanding the role of SVs in patient survival may unveil novel insights into the complex genetic architecture of neurodegenerative disorders and aid in the identification of treatment-specific patient subgroups.

To support the clinical trial analysis and interpretation of DNA sequencing data, we have developed VariantSurvival, a clinical genetic framework. VariantSurvival uses NGS data and other phenotypic inputs such as age, sex, and other clinical information to evaluate the associations between genomic variants and survival outcomes. The software links multiple genetic information from neurological and psychiatric conditions to known genes in the disease of interest. Users can upload data of genomic structural variants, select from a list of genes, and view the results in the form of survival functions, Cox regression tables, and other visualizations and data tables. VariantSurvival is user-friendly and accessible to clinicians and other users without a background in genetics.

## 2 Results

We present VariantSurvival, a new R package, to launch a shiny dashboard (Chang and Ribeiro, 2021; Chang et al., 2022) to visualize and summarize genotype–treatment response for selected medical conditions and target genes. The Supplemental Material of this work contains the Methods section, which describes the statistical models, implementation, and usability in detail. Here, we summarize the application and examine a use case.

Our results demonstrate that VariantSurvival effectively accommodates the analysis of non-implicated genes in the context of neurodegenerative diseases. Despite its primary focus on structural variants, the tool yielded meaningful insights into the impact of the selected non-implicated gene on survival outcomes in the chosen disease dataset. This confirms the tool’s flexibility and its potential to uncover previously unrecognized associations between genes and survival, even when those genes are not conventionally linked to the disease under investigation.

### 2.1 Usage and data input

VariantSurvival requires two types of formatted input: metadata of the clinical trials (patient ID, clinical trial groups, survival status, and time to event) and structural variant information of all individuals participating in the clinical trial.

The set of structural variants across all individuals of the clinical trial is expected to be formatted in the standard variant call format (VCF) and requires gene annotation for each variant. More precisely, VariantSurvival requires a multi-sample VCF file where each variant record is annotated with gene identifiers according to the Ensembl database (Cunningham et al., 2022). For a given target gene selected by the user, VariantSurvival creates a tally summarizing the number of structural variants affecting the target gene for each individual.

The second mandatory input for VariantSurvival is the metadata. Provided that there is a non-empty set of individuals presenting SVs in the target gene, the application proceeds with associating the corresponding clinical trial group labels with all entries (individuals) of the tally. At this point, VariantSurvival computes and visualizes the survival function of Kaplan–Meier product limit estimates (Kaplan and Meier, 1958). Depending on the extent of the provided metadata, the user can select additional features to be used as independent covariates for a Cox regression model that tests whether these features are associated with the time to event.

Further instructions on how to generate and format the input data, test data in order for the user to familiarize themselves with the dashboard, and instructions on how to install the R package and run the application are available at the GitHub repository of VariantSurvival.

### 2.2 The dashboard interface

The VariantSurvival shiny application is designed as a standalone dashboard. Its functionality is grouped into three primary web browser-like tabs.

#### 2.2.1 Select Target Gene tab

**FIGURE 1:**
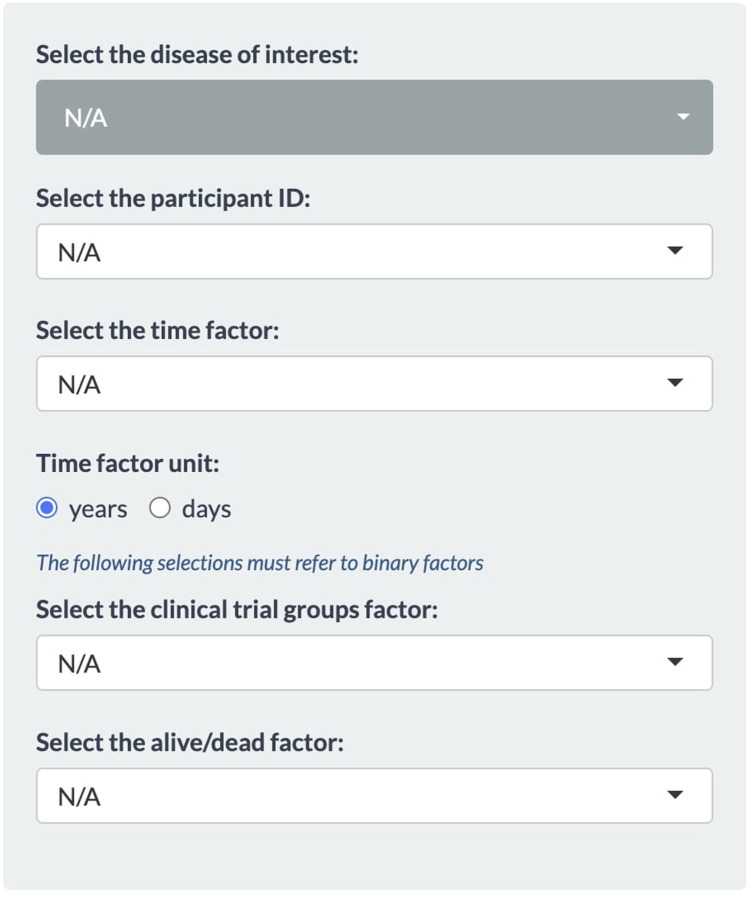
Panel of dropdown menus within the Select Target Gene tab.

The first tab of the application that is on display by default is *Select Target Gene*. This tab is primarily designed for user interaction and feature selection from the metadata. On the left panel (Figure 1), the user must select from multiple dropdown menus. The topmost dropdown menu provides a selection of diseases. The initial release of the application is focused on neurodegenerative diseases. The full list of diseases can be found in Section 1.2 of the Supplementary Material. When selecting a disease, the dashboard immediately displays a list of genes that have previously been associated with the disease based on the ClinGen database (Rehm et al., 2015). To continue with an analysis of a certain gene, the user must select the metadata first. This way VariantSurvival can connect the list of genes with a clinical trial group. For the remaining dropdown menus, the user must select the corresponding features from the metadata sheet. Mind that the time factor unit must be set accordingly. The features selected for the trial group factor and dead/alive factor must be binary features in the metadata. An example of feasible features is shown in the section *Exemplary case* and in the online material.

Once all dropdown menus are set, the *Summary* panel is filled with data. At the top, a table summarizes the number of patients whose genomes contain SVs in any of the genes associated with the selected neurological condition. A search bar aids the lookup for target genes of interest. At the bottom, the user can select one certain gene of interest associated with the disease. For the selected gene, the panels underneath display quantities, histograms, and sample IDs of patients whose genomes contain SVs in the corresponding gene region. Moreover, a histogram in the *Structural Variants Distribution* panel shows the quantity of SVs in each patient and treatment group.

#### 2.2.2 Kaplan–Meier tab

The second tab of the dashboard is *Kaplan*–*Meier* and displays survival functions according to the Kaplan–Meier product limit estimator (Kaplan and Meier, 1958).

The Kaplan–Meier tab consists of two data panels. The *Null model* panel shows the survival function of all data points, presenting a baseline survival probability over time. The *Multiple model* panel shows the data separated by the treatment group and the presence or absence of structural variants in the patients’ target gene.

The small red cogwheel in the top left corner, visible only on the Multiple model panel, provides additional settings to fine-tune data visualization. For instance, the user can constrain the minimum or maximum number of observed variants in a target gene, add confidence intervals, or display a risk table that summarizes the counts of each individual group. Below the plot window, the user can also inspect the life table, listing all numeric values corresponding to the function graphs in detail. The life table is separated into two panels: one for the group of patients with SVs and one for the group of patients without SVs in the target gene.

#### 2.2.3 Cox regression tab

The third tab of the dashboard is *Cox regression*. In this tab, the user can select numerical and categorical covariates for a Cox proportional hazards model (Cox, 1972). Subsequently, its effect parameters are estimated, and the hazard ratio is reported for every selected covariate. Similar to the Kaplan–Meier product limit estimates, the results are listed and separated into a *Standard model* and a *Multiple model* using all patient data or the data are separated by the presence or absence of structural variants in the patients’ target gene. Section 1.1 of the Supplementary Material contains a mathematical description of the SV aware of the Cox proportional hazards model.

### 2.3 Exemplary case

To demonstrate the functionality and usability of VariantSurvival, we present an exemplary case of investigating one specific target gene in a cohort of anonymized clinical samples. The participants in the clinical trial are separated into a placebo group and treatment group, where the members of each group were either not exposed to a drug treatment or received the drug, respectively. DNA samples from all patients in the study were extracted and sequenced using an Illumina NGS assay. A genomic dataset of paired-end short-read WGS data was provided for each patient. For each genomic dataset in this case study, we generated a set of predicted SVs, detected using the Illumina ExpansionHunter tool (Dolzhenko et al., 2017). In addition, all individual sets of SVs were merged into one joint set of SVs of the entire cohort. Each structural variant in the joint set of SVs is annotated with Ensembl gene identifiers if it matches a corresponding gene region. The online material of VariantSurvival contains documentation on how to replicate this data preparation.

**FIGURE 2:**
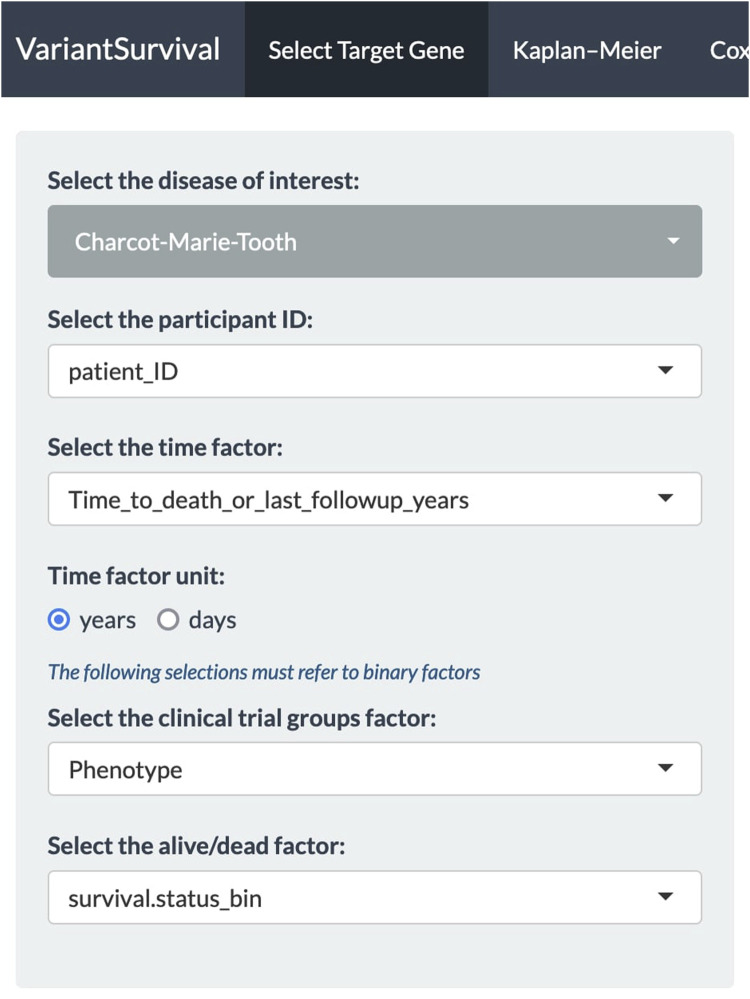
Panel of dropdown menus with selected features to investigate the Charcot–Marie–Tooth syndrome.

In our case, we investigated the *SETX* gene, e.g., as found in association with motor neurone disease (Al Khleifat et al., 2022) or the *Charcot–Marie–Tooth syndrome* (Yoshimura et al., 2019). It should be emphasized that a gene associated with risk does not necessarily imply an effect on survival. First, all the dropdown menus in the Select Target Gene tab are set (Figure 2). We select the Charcot–Marie*–*Tooth syndrome and select the feature “patient_ID” from the metadata sheets as participant ID. The time factor for the Kaplan–Meier product limit estimator is selected as the “time to death or last follow-up in years” from the metadata features. The time factor unit is set to years accordingly. The binary metadata features “Phenotype” and “survival.status_bin” are selected for the clinical trial group factor and alive/dead factor, respectively.

**FIGURE 3:**
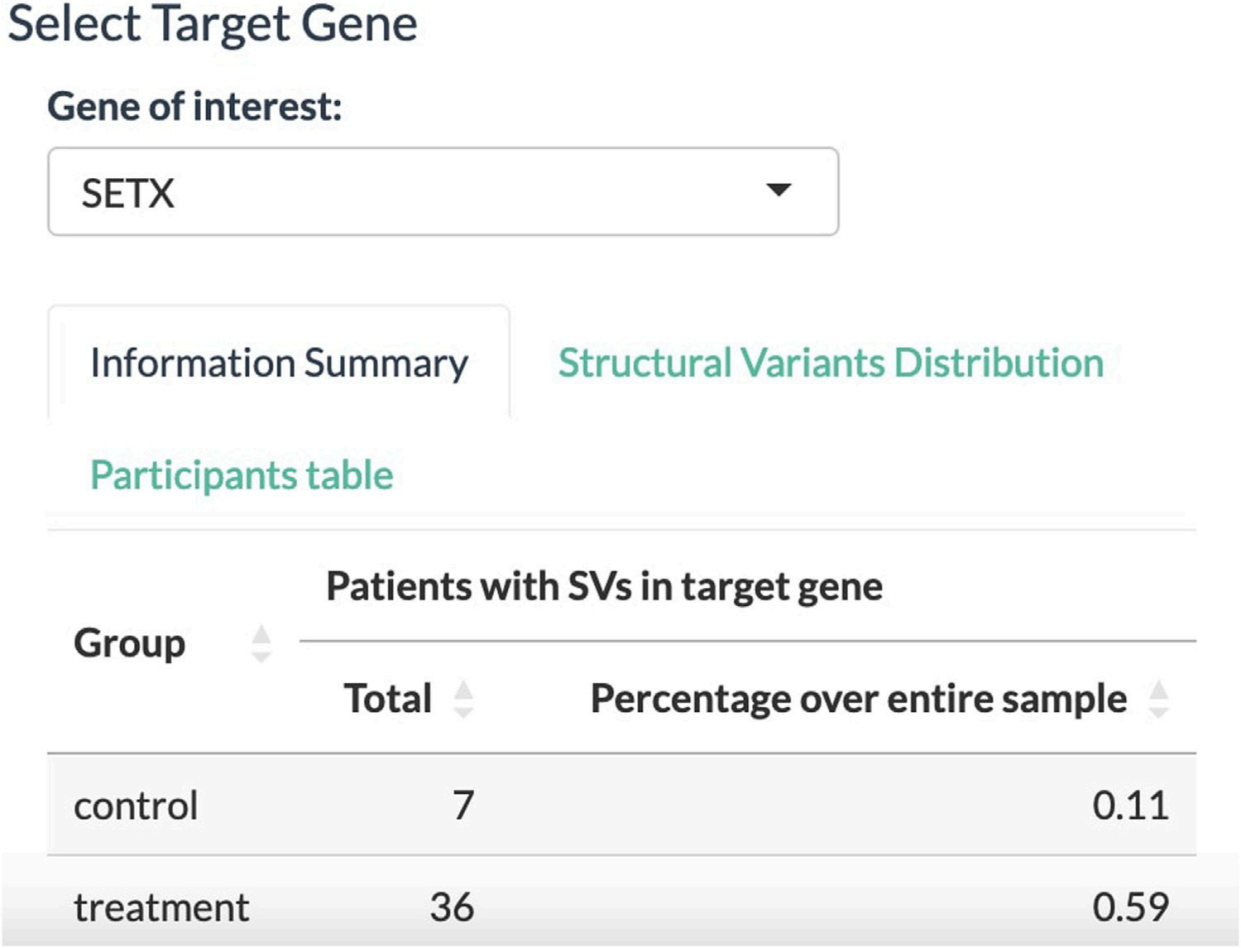
Select Target Gene panel to select a gene associated with the disease of interest. The tally displays the absolute number and relative proportion of participants affected by SVs in the target gene for the placebo and treatment groups.

With all the dropdown menus properly set, the Select Target Gene tab displays a table of associated biomarkers. From the column of gene symbols, we can choose and focus on one gene and select it in the dropdown menu below. In this exemplary case, the *SETX* gene is selected (Figure 3). The information summary reports 43 participants from the clinical trial that carry a variant allele in the *SETX* gene, including seven participants from the placebo group and 36 participants from the treatment group.

**FIGURE 4:**
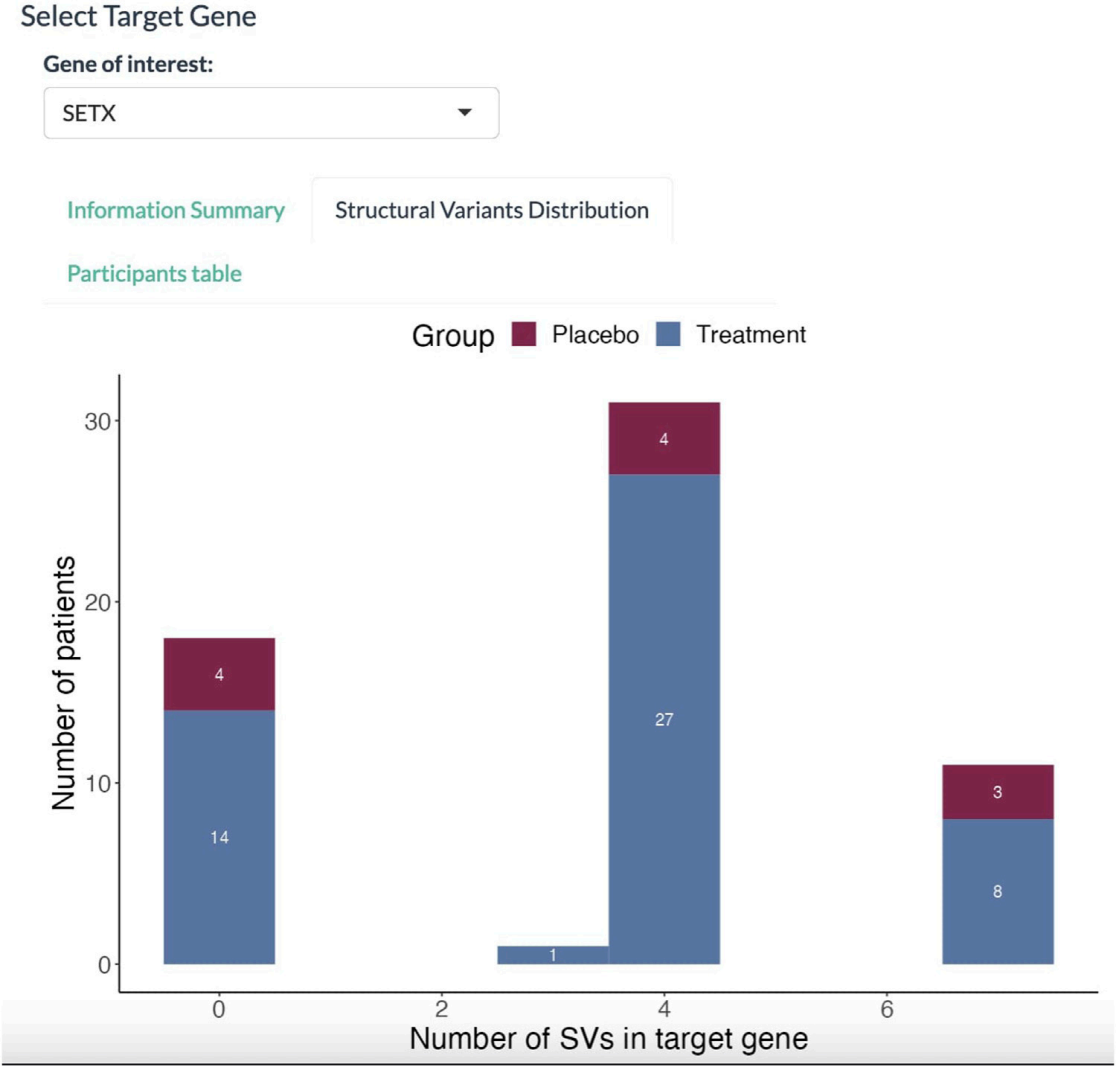
Histogram of participants affected with SVs. The abscissa denotes the number of SVs in the target gene. The proportion in red denotes the placebo group; the proportion in blue denotes the treatment group.

In the Structural Variants Distribution panel (Figure 4), the dashboard displays a histogram with the number of patients for each SV found in the *SETX* gene. Here, 36 out of 50 participants in the treatment group have at least three SVs in the *SETX* gene.

**FIGURE 5:**
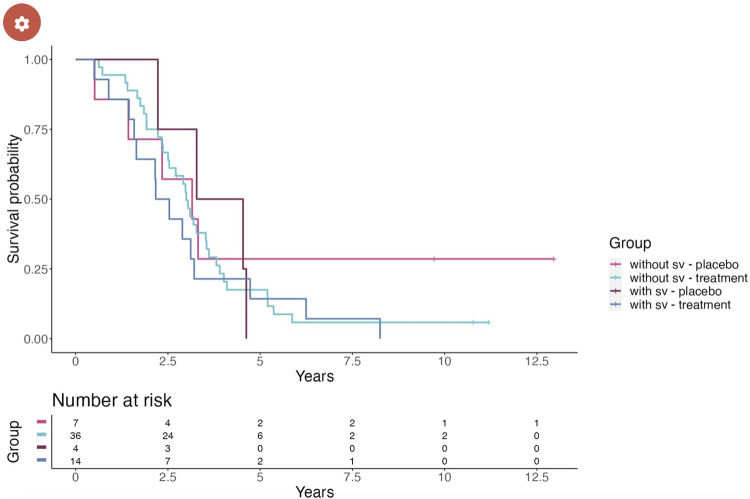
Survival functions according to the Kaplan–Meier product limit estimates. The four functions represent participants of the placebo group not affected by SVs in the target gene (pink), participants of the placebo group affected by SVs (teal), participants of the treatment group not affected by SVs (purple), and participants of the treatment group affected by SVs (blue).

The second tab of the dashboard, i.e., Kaplan–Meier, displays the survival functions (Figure 5). For both the placebo and treatment groups, two survival functions are computed for the subgroups of participants as either carrying or not carrying SVs in the selected *SETX* gene. The graphs indicate two factors of longevity according to the clinical trial data. First, from the participants of the placebo group who were affected by SVs in the target gene, none survived longer than 5 years after their medical condition was reported. In contrast, the corresponding treatment group contains participants (three as listed in the table) surviving longer than 5 years. Second, participants in the placebo group that are not affected by SVs in the target gene have a higher survival probability of more than 5 years after the condition was reported than the participants in the placebo group affected by SVs in the target gene. Moreover, a minor drop in treatment efficiency can be observed between the treatment groups. Altogether, the observations from the data indicate a positive response to treatment compared to the baseline placebo effect as well as an obtrusive effect of SVs in the target gene on the response to treatment.

**FIGURE 6:**
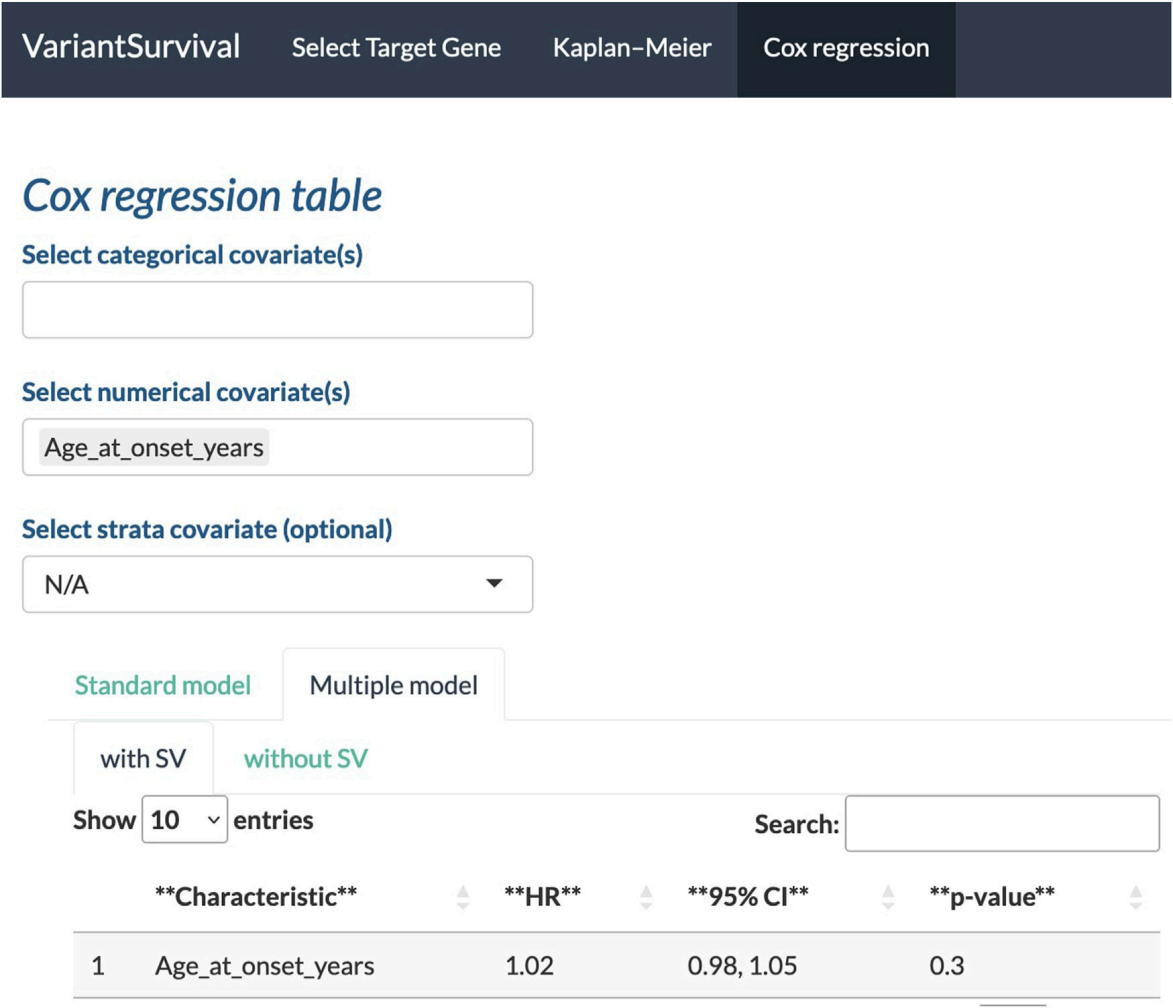
Age at onset in years selected as a feature to determine the Cox proportional hazards ratio.

With the Cox regression tab, we can investigate the impact of the selected features on survival. Here, we investigate whether the *age at onset* is an explanatory numerical covariate. As shown in Figure 6, the age at onset has a hazard ratio of 1.02 in the group of participants with SVs affecting the target gene. For a continuous covariate, the hazard ratio indicates the change in the risk of death with a change in the reference unit by 1 (Zwiener et al., 2011). Here, the reference unit is time in years, i.e., an increase by 1 year in age at onset increases the risk of death by two percent.

It is important to mention that this particular exemplary case is presented on simulated data (Gaastra et al., 2016) to solely demonstrate the functionality of VariantSurvival. The treatment groups of participants were assigned to simulate a successful clinical trial. The numbers, figures, and data shown in this section should not be used for medical recommendations or decision-making.

## 3 Discussion

### 3.1 Summary

VariantSurvival is a lightweight and user-friendly dashboard that allows the exploration of the prognostic potential of genes and gene sets in a broad range of neurological conditions. This will assist in developing markers to predict treatment response in clinical trials that might lead to the identification of specific treatment groups.

The ability of VariantSurvival to assess the effects of non-implicated genes on survival outcomes in neurodegenerative diseases highlights its adaptability and broader utility. Researchers can use the tool to explore novel gene–survival relationships, potentially leading to new insights and hypotheses in the field of neurodegenerative research. This versatility makes VariantSurvival a valuable resource, not only for studying structural variants but also for investigating various gene contributions to survival in diverse disease contexts.

### 3.2 Limitations

The current version (v0.1.1) of the application is limited to neurodegenerative diseases. Furthermore, the dashboard is currently designed and tailored to solely process large genomic structural variants from input data.

### 3.3 Outlook

Future directions for the extension of VariantSurvival include the support for additional types of variants (SNVs and indels) and omics data (methylation assay, RNA data, and the retrovirus database (Pačes et al., 2002)). Furthermore, our aim is to extend the scope of VariantSurvival to address other types of medical conditions (cancer, psychological disorders, etc.). Together with an extended spectrum of medical conditions, we aim to automate the updates of target gene lists and to move on from manual batch updates.

## Data Availability

The original contributions presented in the study are included in the article/Supplementary Material; further inquiries can be directed to the corresponding author.
