# Supplementary material for: VariantSurvival: a tool to identify genotype–treatment response
Source: Front Bioinform. 2023 Oct 11;3:1277923. doi: 10.3389/fbinf.2023.1277923 (PMC10598652; doi:10.3389/fbinf.2023.1277923)
Supplement: Supplementary file 1 [file DataSheet1.pdf]

Supplementary Material of

VariantSurvival: A tool to identify genotype-treatment response

Thomas Krannich[1†], Marina Herrera Sarrias[2†], Hiba Ben Aribi[3†], Moustafa Shokrof[4†], Alfredo Iacoangeli[5], Ammar Al-Chalabi[5], Fritz J Sedlazeck[6], Ben Busby[7], Ahmad Al Khleifat[5*]

[1]Genome Competence Center (MF1), Robert Koch Institute, 13353 Berlin, Germany
[2]Computational Mathematics Division, Department of Mathematics, Stockholm University, Stockholm, Sweden
[3]Faculty of Science of Tunis, University El Manar, Tunis, Tunisia
[4]Department of Computer Science, University of California, Davis, CA, USA
[5] Wohl Clinical Neuroscience Institute, King's College London, London SE5 9RX, UK
[6]Human Genome Sequencing Center, Baylor College of Medicine, Houston, TX, 77030, Maurice
[7] DNAnexus, Mountain View, CA, USA

[†]These authors contributed equally to this work and share first authorship.

*** Correspondence:**
Ahmad Al Khleifat
Ahmad.al_khleifat@kcl.ac.uk

1 Methods

1.1 Structural variants determine subgroups for the Cox proportional hazard model

In order to compute the Cox proportional hazard ratio for our multiple model (Figure 6) we separate the set of participants P into the two subgroups S^+ of individuals with structural variants in the target gene and S^- of individuals that do not have a structural variant in the target gene.
Hence, $S^+ \cup S^- = P$. Then, the proportional hazards for each subgroup have the form

$$h^- (t \,|X_i\,, i \in S^-) \;=\; h_0(t) \;*\; exp(\beta_1 X_{i1} + \ldots + \beta_n X_{in}) \text{ and}$$

$$h^+ (t \,|X_i\,, i \in S^+) \;=\; h_0(t) \;*\; exp(\beta_1 X_{i1} + \ldots + \beta_n X_{in}) \,.$$

The vector $X_i = (X_{i1} + \cdots + X_{in})$ is the vector of covariates for individual i, the input t is a timepoint in the study, $h_0(t)$ is the baseline hazard, the β are the effect coefficients that are to be estimated with a Maximum-Likelihood estimation and n is the number of covariates.

Currently, the Cox regression tab of VariantSurvival provides input fields for two types of covariates, categorical and numerical covariates.

1.2 The shiny dashboard and R package

Development. The Variantsurvival package was developed using the devtools package (Wickham, Hester, Chang, et al., 2022) in the R programming language and documented with the Roxygen2 package (Wickham, Danenberg, Csárdi, et al., 2022). VariantSurvival consists of a single function that serves as a shiny app (Chang et al., 2022) to analyze genotype-treatment response. The app interface was created using various R packages, including shiny (Chang et al., 2022), shinydashboard (Chang & Ribeiro, 2021), shinyjs, shinyWidgets, shinythemes, shinycssloaders, and DT (Xie et al., 2022). Additionally, other R packages, such as ggplot2 (Wickham, 2016), dplyr (Wickham, François, Henry, et al., 2022), tidyverse (Wickham et al., 2019), vcfR (Knaus & Grünwald, 2017), readr (Wickham, Hester, & Bryan, 2022), and readxl (Wickham & Bryan, 2022), were employed for data manipulation. Statistical analysis was carried out using the survival (T. Therneau, 2022), survminer (Kassambara et al., 2021), lubridate (Grolemund & Wickham, 2011), gtsummary (D. Sjoberg et al., 2021) and the ggsurvfit (D. D. Sjoberg, 2022) R packages.

The disease-gene association data was collected from the ClinGen database (Rehm et al., 2015) and categorized by syndromes, with the complete list available in the project's GitHub repository.

List of supported neurological disorders. At the time of issuing this manuscript, the VariantSurvival (v0.1.0) package initially supports the following neurological disorders:

- Amyotrophic Lateral Sclerosis Spectrum Disorders
- Brain Malformations
- Cerebral Palsy
- Craniofacial Malformations
- Epilepsy
- Glaucoma and Neuro-Ophthalmology
- Intellectual Disability and Autism
- Leigh syndrome
- Parkinson disease
- Rett and Angelman-like Disorders
- Charcot-Marie-Tooth

This list is subject to batch updates with diseases to target gene associations in the ClinGen database.

Installation. VariantSurvival can be downloaded and installed using an R environment and the package development tool devtools (Wickham, Hester, Chang, et al., 2022) using the following command lines in R:

```
>library(devtools)
>devtools::install_github("collaborativebioinformatics/VariantSurvival/VariantSurvival_package")

>library(VariantSurvival)
>VariantSurvival::VariantSurvival(vcffile="myVariants.vcf", metadatafile= "myMetadata.xlsx")
```

where *myVariants.vcf* is a VCF file with gene annotation per variant record and *myMetadata.xlsx* is a metadata sheet. Details on the metadata features can be found in the online material (see Data Availability Statement). For simply testing the dashboard we integrated some demo data. The user can access the demo data by replacing the call to the VariantSurvival function above with:

```
>VariantSurvival::VariantSurvival(demo=TRUE)
```

References

- Grolemund, G., and Wickham, H. (2011). Dates and times made easy with lubridate. J. Stat. Softw. 40 (3), 1–25. doi:10.18637/jss.v040.i03
- Kassambara, A., Kosinski, M., Biecek, P., and Scheipl, F. (2021). survminer: Drawing Survival Curves using “ggplot2“.
- Knaus, B. J., and Grünwald, N. J. (2017). Vcfr: A package to manipulate and visualize variant call format data in R. Mol. Ecol. Resour. 17 (1), 44–53. doi:10.1111/1755-0998.12549
- Sjoberg, D. D. (2022). ggsurvfit: Easy and flexible time-to-event figures.
- Sjoberg, D., Whiting, K., Curry, M., Lavery, J., and Larmarange, J. (2021). Reproducible summary tables with the gtsummary package. R J. 13, 570–580. doi:10.32614/RJ-2021-053
- Therneau, T. (2022). A package for survival analysis in R.
- Wickham, H. (2016). ggplot2: Elegant graphics for data analysis. Springer-Verlag New York. https://ggplot2.tidyverse.org.
- Wickham,H., Averick,M.,Bryan, J.,Chang,W.,McGowan,L.D., François,R., et al. (2019). Welcome to the tidyverse. J. Open Source Softw. 4 (43), 1686. doi:10.21105/joss.01686
- Wickham, H., and Bryan, J. (2022). readxl: Read excel files.
- Wickham, H., Danenberg, P., Csárdi, G., and Eugster, M. (2022). roxygen2. Line Documentation for R.
- Wickham, H., François, R., Henry, L., and Müller, K. (2022). Dplyr: A grammar of data manipulation.
- Wickham, H., Hester, J., and Bryan, J. (2022). readr: Read rectangular text data.
- Wickham, H., Hester, J., Chang, W., and Bryan, J. (2022). devtools: Tools to make developing R packages easier.
- Xie, Y., Cheng, J., Tan, X., Allaire, J. J., Girlich, M., Ellis, G. F., et al. (2022). DT: wrapper of the JavaScript library “DataTables“.
